# Reply to Mr. Syer

**Published:** 1801-03

**Authors:** 


### Reply to Mr. Syer.

In your laſt Number, (No. xxiv. vol. v. p. 170) a gentleman of the name of Syer has done me the honour to publiſh ſome Remarks on a paper of mine, which was inſerted in a former Number of your Journal, and contained an account of a caſe of Monſtroſity that had recently occurred to me. Whether my reaſoning be juſt or not, or my opinions well or ill founded, your readers muſt determine; but as Mr. S. appears to have been actuated by laudable motives, I ſhall, in this inſtance, ſay a few words in reply. And,

1. I muſt requeſt Mr. S. to re-peruſe the paper upon which he has beſtowed his comments, and he will diſcover, that in deſcribing the appearances, he has miſtated my words, no doubt unintentionally; but the miſtake would have been avoided by tranſcribing the paſſage.

2. He objects to my firſt poſition drawn from that caſe, namely, namely, "that nervous influence is not at all neceſſary to the growth of the fœtus in utero;" which, he ſays, "is anſwered by the *universal* exiſtence of a brain and nerves in the fœtus;" and yet he admits, at the concluſion of the ſame ſentence, that that caſe furniſhes "a ſolitary exception."

3. He likewiſe objects to my other poſition, which is, "that the fœtus in utero does not poſſeſs ſenſation;" and ſtates, that this "is not leſs liable to animadverſion" than the former. I had certainly to learn, that a concluſion upon a point of phyſiology, drawn with every appearance of ſincerity, even if erroneous, could be deſerving of cenſure. I confeſs it has ever appeared to me, both more ſafe and more philoſophical to deduce directly from appearances, though the interference may not accord with a pre-conceived theory, than to object on hypothetical grounds, becauſe "Nature appears to have intended."

There was no veſtige of optic nerve. The recurrents were not traced.

I ſhall be very glad to ſhow the preparation to Mr. Syer, ſhould he come this way.

